# Eleventh International Foamy Virus Conference—Meeting Report

**DOI:** 10.3390/v8110318

**Published:** 2016-11-23

**Authors:** Florence Buseyne, Antoine Gessain, Marcelo A. Soares, André F. Santos, Magdalena Materniak-Kornas, Pascale Lesage, Alessia Zamborlini, Martin Löchelt, Wentao Qiao, Dirk Lindemann, Birgitta M. Wöhrl, Jonathan P. Stoye, Ian A. Taylor, Arifa S. Khan

**Affiliations:** 1 Unité d’Épidémiologie et Physiopathologie des Virus Oncogènes, Institut Pasteur, 75015 Paris, France; antoine.gessain@pasteur.fr; 2 Centre National de la Recherche Scientifique (CNRS), UMR3569, 75015 Paris, France; 3 Department of Genetics, Universidade Federal do Rio de Janeiro, 21949-570 Rio de Janeiro, Brazil; masoares@biologia.ufrj.br (M.A.S.); andre.santos@ufrj.br (A.F.S.); 4 Oncovirology Program, Instituto Nacional de Câncer, 20231-050 Rio de Janeiro, Brazil; 5 Department of Biochemistry, National Veterinary Research Institute, 24-100 Pulawy, Poland; magdalena.materniak@piwet.pulawy.pl; 6 Institut Universitaire d’Hématologie, Hôpital Saint-Louis, Université Paris Diderot, Sorbonne Paris Cité, INSERM U944, CNRS UMR 7212, 75010 Paris, France; pascale.lesage@inserm.fr (P.L.); alessia.zamborlini@univ-paris-diderot.fr (A.Z.); 7 Conservatoire National des Arts et Métiers, Laboratoire de Pathologie et Virologie Moléculaire, 75003 Paris, France; 8 Department of Molecular Diagnostics of Oncogenic Infections, Research Program Infection, Inflammation and Cancer, 69120 Heidelberg, Germany; m.loechelt@dkfz.de; 9 Key Laboratory of Molecular Microbiology and Technology, Ministry of Education, College of Life Sciences, Nankai University, Tianjin 300071, China; wentaoqiao@nankai.edu.cn; 10 Institute of Virology, Medical Faculty “Carl Gustav Carus”, Technische Universität Dresden, 01307 Dresden, Germany; dirk.lindemann@tu-dresden.de; 11 University of Bayreuth, Department of Biopolymers, 95447 Bayreuth, Germany; birgitta.woehrl@uni-bayreuth.de; 12 Francis Crick Institute, London NW1 1AT, UK; jonathan.stoye@crick.ac.uk (J.P.S.); ian.taylor@crick.ac.uk (I.A.T.); 13 Laboratory of Retroviruses, Division of Viral Products, OVRR, CBER, U.S. Food and Drug Administration, Silver Spring, MD 20993, USA; arifa.khan@fda.hhs.gov

**Keywords:** foamy virus, cross-species virus transmission, zoonosis, restriction factors, immune responses, FV vectors, virus replication, latent infection

## Abstract

The Eleventh International Foamy Virus Conference took place on 9–10 June 2016 at the Institut Pasteur, Paris, France. The meeting reviewed progress on foamy virus (FV) research, as well as related current topics in retrovirology. FVs are complex retroviruses that are widespread in several animal species. Several research topics on these viruses are relevant to human health: cross-species transmission and viral emergence, vectors for gene therapy, development of antiretroviral drugs, retroviral evolution and its influence on the human genome. In this article, we review the conference presentations on these viruses and highlight the major questions to be answered.

## 1. Introduction

The biennial series of Foamy Virus (FV) conferences represents an important international forum devoted to all aspects of foamy virus research. The 11th conference took place in Paris, following an uninterrupted series of meetings since 1994 in London [1]. Some participants were interested in the unique features of these viruses, as typified by the late Axel Rethwilm. Others were attracted by the opportunities to gain knowledge relevant to human health offered by the same unique properties of these viruses. Key facts on FVs were presented to the multidisciplinary audience during review talks and are summarized in Table 1.

Molecular studies have been driven by the development of gene therapy vectors. New data on restriction factors and/or use of foamy virus vectors for vaccine delivery will hopefully increase the benefits gained from basic science research on FVs. In vivo studies in animals and ex vivo studies of human samples are critically lacking. Nevertheless, recent in vivo data has challenged established dogmas, such as the restricted replication of FVs in the oral cavity or their total absence of pathogenicity. The diversity of results obtained from different animal species, or from humans infected with strains from different simian species illustrated knowledge gaps regarding the in vivo sites of viral replication, the initiation, maintenance and reversal of latency and mechanisms of immune control. Table 2 presents the key unanswered questions on FVs and the following section summarizes the scientific presentations.

## 2. Summary of Scientific Sessions

### 2.1. Foamy Virus Infection of Animal Hosts

#### 2.1.1. Background and Review Talk

FVs infect a wide range of mammals, including primates, felids, equines, ruminants and bats. Marcelo Soares reviewed recent studies that reported endogenous FV sequences in sloths, the Cape golden mole, prosimians and the fossil fish coelacanth, suggesting an FV–host relationship of over 400 million years [2]. The prevalence of FV infection varies widely between animal families, communities and between wild and captive animals, ranging from 15% to 75% in the former and up to 100% in the latter.

The described routes of FV transmission include biting and grooming [3], as well as a single report describing vertical transmission in chimpanzees [4]. In experimental animals, blood transfusion and organ transplantation transmitted FV infection [5]. Virus transmission through saliva and other airway fluids is consistent with reports showing that FV replication occurs primarily in the oropharyngeal tract and lung tissues, where viral RNA is found [6]. FV DNA has been observed in other tissues and organs, such as blood, kidneys, liver, brain and the gastrointestinal lymphoid tissue [7].

There is poor evidence of any pathogenic potential of FV in their hosts so far, including in humans. A single study reported histopathological findings in the kidney of FV-inoculated domestic cats indicative of syncytia formation [8]. In a more recent study conducted with simian immunodeficiency virus (SIV)-infected rhesus macaques, Choudhary and colleagues have shown that those coinfected with the simian FV (SFV) progressed faster to the acquired immune deficiency syndrome (AIDS), had higher SIV viral loads and accelerated CD4^+^ T-cell depletion when compared to animals singly infected with SIV [9]. This raises the possibility that FV can act as an opportunistic pathogen, modulating the pathogenic impact of other infections.

#### 2.1.2. Summary of the Presentations

At the Eleventh International Foamy Virus Conference held in Paris, interesting presentations were delivered that contribute to, or even challenge the issues discussed above, regarding FV tropism and pathogenesis. Marcelo Soares and colleagues reported the use of different methods for FV detection in non-human primates (NHPs), including noninvasive sampling, and were able to generate full-length FV sequences and isolates. In addition, information on viral infection of novel species contributes to the understanding of the origin and dissemination of FV in animals.

In another presentation, Magdalena Marterniak-Kornas and colleagues surveyed the FV tissue tropism of cows experimentally inoculated with the bovine FV (BFV). They searched for BFV RNA in different tissues of euthanized calves and also in sorted lymphocyte subpopulations isolated from peripheral blood mononuclear cells (PBMC). While they found BFV RNA in most analyzed tissues, only one of four calves tested positive in the saliva.

A talk on the potential opportunistic effect of FV in animals was given by André Santos. He and his colleagues have compared infected domestic cats of three different categories, feline FV (FFV)^+^, feline leukemia virus (FeLV)^+^ and FFV/FeLV-coinfected animals, with respect to the viral load (VL) of each virus in both PBMC and buccal swabs. They showed that FFV VL in the buccal swabs of FFV/FeLV-coinfected animals was 1000 fold higher than that of FFV-monoinfected cats, but no differences in the PBMC were seen. FeLV VL did not differ between FeLV-monoinfected and FFV/FeLV-coinfected cats, nor did FeLV-specific clinical signs between the two groups.

#### 2.1.3. Perspectives

A number of questions still remain unanswered with respect to FV host and tissue ranges, and to their pathogenic nature in animals. The idea that oropharyngeal tissues are the major, or even the unique sites of FV replication might be an imprecise generalization. Further studies conducted in different models and with experimental inoculation to enable true viral dynamics follow-up are warranted. With respect to the pathogenic potential of FV, either per se or in the context of coinfection with other pathogens, additional observational and experimental scenarios deserve investigation to elucidate their actual impact on animal and human health.

### 2.2. Cross-Species Transmission of Foamy Viruses to Humans

#### 2.2.1. Background and Review Talk

Interspecies transmission of SFVs from monkeys or apes to humans is a relatively frequent event in populations of persons at risk. These include occupationally exposed individuals working in zoos, primate research centers and animal care facilities, hunters of monkeys and apes in Central Africa and people in contact with monkeys in Southeast Asia, where monkeys and humans are sympatric in certain highly populated areas. Antoine Gessain reviewed the past and current works on human infection with zoonotic FVs of simian origin. Studies in Europe, USA, Central Africa and Asia have clearly demonstrated such cross-species transmission [10]. Important differences exist between Africa and Asia concerning the interface between NHPs and humans, which can lead to transmission of SFVs. He then focused his review on the studies performed in his unit with collaborators in Cameroon and Gabon. A series of more than 60 infected individuals, mostly hunters of apes has now been reported. The molecular characterization of five zoonotic isolates by complete sequence analysis has shown the presence of natural polymorphisms without any evidence of viral adaptation. Despite undetectable viral RNA, viral DNA was present in both blood and saliva cells from 14 individuals infected with a gorilla SFV, suggesting latent infection. Lastly, in persons infected with a gorilla strain, CD8^+^, CD4^+^ and B lymphocytes were the major cellular targets for SFV in the peripheral blood [11].

#### 2.2.2. Summary of the Presentations

Very little is known about the potential zoonotic transmission of SFVs from New World monkeys (NWM) to humans. Marcelo Soares conducted a longitudinal, prospective study of 56 workers occupationally exposed to NWM in Brazil. Using a Western Blot (WB) assay containing NWM SFV antigens, ten persons tested positive. All of these seropositive workers reported at least one incident involving NWM, including bites. Using different NWM-specific PCR, no viral DNA found in the blood or the oral swabs from WB-reactive workers. SFV antibody clearance was found in four workers during a three year follow-up period. These data suggest that NWM SFV can be transmitted to occupationally-exposed humans and can elicit specific humoral immune responses, but without evidence of persistent infection in peripheral blood. Interestingly, quite similar results were obtained in a recently published work by a US team [12].

Léa Richard reported findings concerning the genetic diversity of the *env* gene of gorilla and chimpanzee SFV strains infecting NHPs and hunters from Central Africa. The complete *env* gene or the fragment coding for the surface protein (SU) were amplified from blood DNA of a series of 40 individuals from Cameroon or Gabon infected with a gorilla or chimpanzee SFV. Phylogenetic analyses revealed the existence of two *env* variants amongst both the gorilla and chimpanzee SFV strains that were present both in humans and NHPs. These variants differed greatly (>30% variability at the nucleotide level) in a 753-bp-long region located in the receptor-binding domain of SU, whereas the remainder of the *env* gene was largely conserved. Analysis of recombination, by different methods, suggests that the variants emerged through recombination events between different strains, although not all parental strains could yet be identified [13]. Preliminary data provided evidence for dual infection with strains from different genotypes in gorillas, chimpanzees and humans.

SFVs are efficiently transmitted to humans and establish persistent infection. Neither a pathogenic effect nor human-to-human transmission has yet been reported, suggesting efficient immune control of this retrovirus. Caroline Lambert presented data on SFV-specific neutralizing antibodies in infected individuals. Plasma samples from infected hunters were tested for their ability to neutralize two gorilla isolates belonging to the two genotypes described above. Neutralizing antibodies were detected in 40 out of 44 infected hunters. Both genotype-specific (*n* = 24) and cross-reactive (*n* = 16) neutralization were observed. For half of individuals with cross-neutralizing antibodies, infection with two SFV strains was shown. Most plasma samples from gorilla SFV-infected individuals were able to neutralize chimpanzee SFV strains, showing that neutralizing antibodies target conserved epitopes.

#### 2.2.3. Perspectives

There are three main questions that remain to be answered about human infection with SFVs. What are the distribution and the magnitude of such zoonotic infections in Africa, Asia, and America? Are there biological abnormalities and/or clinical symptoms associated with SFV infection in humans? What are the roles of restriction factors on viral replication and virus transmissibility in infected human hosts?

### 2.3. Integration and Endogenous Foamy Viruses

#### 2.3.1. Background and Review Talk

Integration of the viral genome is a key step of retroviral infection, which ensures expression of viral genes, and thus production of new progeny viruses, as well as stable maintenance and transmissibility of the viral genetic material. FVs have a remarkably stable and long co-speciation history with their hosts, stretching back more than 400 million years, which allows examination of their evolution across an extended period of time. The two first presentations of this session focused on cellular cofactors involved in the integration process, while the last talk traced the origin of FVs. Integration is performed by the retroelement-encoded integrase and is not a random process. Each retrovirus genus displays a distinct and specific pattern of insertion, which is regulated by viral and cellular factors as well as by local DNA conformation and chromatin structure at the site of integration. A better understanding of the integration site selection process has recently emerged with the advent of next-generation sequencing methods to identify insertion sites, the resolution of the architecture of the prototype FV (PFV) integration machinery [14] and the identification of cellular cofactors involved in the process. In the first keynote lecture of the meeting, Pascale Lesage presented an overview of this process, highlighting how the DNA sequence and chromatin contexts, cellular proteins, and the spatial organization of the nucleus contribute in targeting retroviral integration to specific chromosomal regions. She reviewed recent data on the structural basis of FV integration into nucleosomes [15] and also described how her lab recently identified the AC40 subunit of RNA polymerase III as the cellular factor responsible for the peculiar distribution of the model Ty1 retrotransposon of yeast, which integrates close to Pol III genes [16].

#### 2.3.2. Summary of the Presentations

In his presentation, Dirk Lindemann described an interaction between PFV Gag and cellular polo-like kinase (PLK) proteins, which is important for early steps of viral replication [17]. His laboratory identified a conserved consensus Ser-Thr/Pro (S-T/P) motif within PFV Gag, which is required for the interaction with PLKs. Importantly, this site was phosphorylated within PFV virions. A functional kinase activity and the polo-box domains of PLKs are also necessary for the binding to PFV Gag. Interestingly, PFV infectivity was decreased up to 20-fold by the presence of mutations in the Gag S-T/P motif or by addition of a PLK inhibitor during early phases of PFV infection. Mutated viruses displayed no defect in morphogenesis, particle release, RNA packaging and intra-particle reverse transcription, but showed post-fusion defects resulting in delayed and reduced integration. The integration site distribution profile was subtly altered indicating that the interaction of PFV capsids with host cell PLKs might be required for efficient integration.

Pakorn Aiewsakun described that a simple power–law decay function can recapitulate the FV evolutionary rate dynamics [18] and that this “time-dependent rate phenomenon” (TDRP) is a pervasive evolutionary feature of all viruses [19]. He also presented the comparison of 36 novel amphibian and fish FV-like endogenous retroviruses (FLERVs), which reveals that some of the ray-finned fish FLERV genomes display unique features because they are extremely large (>17 kb) and contain putative accessory genes. Based on the TDRP model, the origin of FLERVs and FVs is comparable to that of their jaw vertebrate hosts, ~455 million years ago, suggesting an ancient marine origin for retroviruses dating back at least to the early Paleozoic Era.

#### 2.3.3. Perspectives

Structural studies of FV integrase-DNA complexes have provided a major breakthrough for understanding the mechanism of retroviral integration. The next issue will be to determine the role of Gag and specific PLK family members in FV integration site selection.

### 2.4. Foamy Virus Replication and Virus-Cell Interaction

#### 2.4.1. Background and Review Talk

Birgitta Wöhrl reviewed the structure and function of the viral enzymes in the FV life cycle. She focused on the substantial differences in the activation pathways of FV Pol-encoded enzymes compared to the situation in orthoretroviruses. For example, there is no release of a free protease moiety but instead protease activation is mediated by binding to the defined genomic RNA sequence protease-activating RNA motif (PARM) and subsequent dimerization of the Pol polyproteins. For an introduction into the topic, see [20,21,22].

#### 2.4.2. Summary of the Presentations

Suzhen Zhang presented data concerning in vitro-selected BFV with a high titer cell-free transmission phenotype. Similar to previous data [23], BFV cell-free transmission can be significantly enhanced by serial cell-free passaging and the authors showed that genetic changes occurred in different regions of the genome. Most importantly, out of five changes in the C-terminal part of Env, the E898K amino acid substitution was especially interesting since it greatly enhanced entry into new target cells without major effects on virus and sub-viral particle release.

Guochao Wei presented deletion analyses of the C-terminus of FFV) Gag. Gross deletions extending into the glycine/arginine-rich C-terminal domain abolished cytosolic capsid assembly as assayed by sedimentation analyses. In addition, Env-dependent particle release was also abrogated upon truncation of Gag proteins while mutations of a highly conserved Y-R-QPQRYG motif in the proviral context only affected particle infectivity. The data may indicate different nucleic acid binding affinities and specificities, for instance for initial capsid assembly compared to generation of fully infectious particles.

Stefanie Richter fascinated the audience with live cell images of PFV entry and membrane fusion using differentially labelled PFV Gag and Env proteins. The very aesthetic movies showed that PFV and macaque SFV (SFVmac) Env proteins exhibit different preferences for entry: while PFV Env allowed entry (as measured by the dissociation of Gag and Env signals) at the plasma membrane and intracellular membranes, plasma membrane entry was not observed for SFVmac Env. These data are in line with inhibitor-based entry studies [24,25].

The following presentations were related to the transcriptional and post-transcriptional regulation of FV gene expression. Taga Lerner presented the outcome of an in vitro selection screen on PFV replication in HEK293 cells. While PFV long terminal repeats (LTR)-directed gene expression and thus production of viral infectivity was extremely low in these cells, variants that took up different parts of the cytomegalovirus-immediate early (CMV-IE) promoter showed high level cell-free infectivity clearly above that previously described [26]. Studies are under way to dissect the effects of the promoter exchange versus additional amino acid substitutions. The selected PFV clone is likely to be an excellent tool for further studies and vector development.

An evolving line of research concerns the characterization of the unique RNA Pol III-transcribed dumbbell-shaped primary microRNAs (miRNAs) of about 120-nt size shown to be encoded by PFV and BFV [27,28]. Wenhu Cao introduced the subject and described promising developments to dissect the functions of the individual hairpins in BFV and to define the virus–host interaction at the post-transcriptional level.

Belete Teferedegne reported on the generation and characterization of cell clones persistently infected with an SFV isolate from rhesus macaque (SFVmmu-K3T) to study virus–host interactions, with a special emphasis on factors controlling virus latency versus replication [29]. A549 cells, which allow SFV latency, were used to develop cell clones positive for SFVmmu-K3T DNA. Co-culture with highly permissive *Mus dunnii* cells and Tas-rescue experiments were conducted to identify clones that could produce replication competent virus indicating an intactness of latent SFV genomes and the validity of the experimental system.

#### 2.4.3. Perspectives

Analyzing the molecular biology of FVs in cell culture systems with a special emphasis on the interaction with the host cell is still an essential, challenging and profitable approach to evaluate the applicability of FVs as gene therapy and vaccine vectors and to understand their biology in the host. Due to the lack of easy-to-handle animal models for FV replication, several studies have to be done in cell cultures. Here, further input based on naturally or experimentally infected animals is required.

### 2.5. Immune Responses to Foamy Viruses

#### 2.5.1. Background and Review Talk

FVs display ubiquitous cellular tropism associated with a strong cytopathic effect in most cell types in vitro. However, these viruses apparently lack pathogenicity in vivo. Their inter- and intra-host genetic stability is high despite replication of the viral genome by a reverse transcriptase prone to a high error rate in vitro. An efficient immune control of FV replication together with their long-term adaptation to their host could explain these two paradoxes. FVs are susceptible to the antiviral action of type I and type II interferons (IFNs), to several restriction factors and to neutralizing antibodies. Neither the induction nor the action of other antiviral mechanisms has been tested against FVs. Florence Buseyne presented an overview of the five phases of the immune response to viruses highlighting knowledge about FV and important missing information: (1) the detection of viral components by innate sensors initiate the immune response. Hematopoietic cell infection with FVs is sensed by Toll-like receptor 7 (TLR7) and leads to IFN-α production [30]; (2) the major outcomes of innate sensing are the synthesis of antiviral molecules and the destruction of infected cells by either inflammasome-induced cell death (pyroptosis) or natural killer cell-mediated cytolysis. FV replication is inhibited by IFN-α, -β, and -γ. Two possible escape mechanisms from the antiviral action of IFNs have been described for FVs: the low lysine content of the Gag protein conferring partial resistance to IFN-α in primary cells and the synthesis of an miRNA inhibiting response to IFN [27]; (3) most steps of the FV life cycle are sensitive to the action of major IFN-induced restriction factors, including the alpha isoform of tripartite motif-containing protein 5 (TRIM5α), apolipoprotein B mRNA editing enzyme, catalytic polypeptide-like (APOBEC), interferon-induced 35 kDa protein (IFP35), TRIM19, and Tetherin [20,31]; (4) regarding the adaptive immune effectors, FV-virus specific T lymphocytes have not been described thus far. Neutralizing antibodies against cell-free FV are detected in most animal and human hosts. Antigenic classes match with host species and *env* genotypes (see session 2 summary, section 2.2) [32]; and (5) last, but not least, antibodies can prevent FV transmission between hosts, as shown in experimental transfusion experiments [33]. Several key steps of virus-induced immune responses remained to be studied for FVs, including the activation of cytosolic nucleic acid sensors, inflammasome and natural killer (NK) cells by FVs, FV-virus specific T lymphocytes, and antiviral activities of antibodies other than neutralization of cell-free virus.

Jonathan Stoye gave the second keynote lecture of the meeting on restriction factors and viral countermeasures, underscoring that the properties of the former constitute the basis for the design of new antiviral molecules. Focusing on capsid-binding restriction factors (CBRFs), Fv1 and TRIM5α, he recapitulated a lengthy saga of key discoveries including the identification of factors controlling in vivo and in vitro susceptibility to infection by retroviruses such as murine leukemia virus (MLV) or human immunodeficiency virus type 1 (HIV-1), CBRF’s genomic structure and regulation as well as their genetic evolution driven by several retroviruses, including a possibly pathogenic FV [34]. The variability of retroviral restriction by diverse CBRFs was key to mapping functional domains. Structural studies of restriction factors showed how their multimerization allows binding to assembled capsid proteins and enhanced the avidity of these interactions [35]. The proposed mode of action of the CBRFs is the formation of a superlattice on viral cores.

#### 2.5.2. Summary of the Presentations

Xiao Xu presented new results on the regulation of the *Nmi* gene. This gene encodes the N-myc interactor, an IFN-inducible protein that sequesters the viral Tas protein in the cytoplasm, thereby inhibiting FV replication [36]. The *Nmi* promoter was isolated and characterized. It was shown to contain an IFN-stimulated response element (ISRE) important for both basal and IFN-induced *Nmi* promoter activation. The IFN regulatory factor 1 (IRF-1) binds to this ISRE and activates the *Nmi* promoter. Xiaomei Hu described the capacity of an IRF to inhibit the PFV replication. This restriction is associated with codon-usage-bias during translation of PFV protein, is active on HIV proteins and requires the activation of downstream target genes. Yingpeng Xie presented data on a TRIM protein that represses viral transcription. A direct interaction with the viral Tas protein was shown, without any effect on its subcellular localization. The mode of action is under study.

Martin Löchelt presented data on the restriction of feline retroviruses by cellular APOBEC3 cytidine deaminases. Both FFV and feline immunodeficiency virus (FIV) encode proteins that inhibit APOBEC3 action. FFV Bet prevents APOBEC incorporation into viral particles whilst FIV-Vif promotes APOBEC proteosomal degradation. Cellular expression of each of these viral antagonists protects the heterologous virus from APOBEC3 restriction. When expressed by a replication competent FFV, the FIV-*Vif* gene functionally replaces the FFV Bet protein in vitro. Upon in vivo infection of cats, both the wild type and the FFV-Vif viruses induced FFV-specific antibodies, but only the wild type virus was persistently detected. These data underscore the importance of such basic science studies for the design of FV-based vaccine vectors. In addition, Martin Löchelt presented experimental evidence that human APOBEC3A expression in genetically engineered HEK293 cells induces mutational signatures known from different cancer entities providing direct evidence that the APOBEC3 cytidine deaminase contributes to the genetic outfit of human cancers.

#### 2.5.3. Perspectives

CBRFs may function as pattern-recognition receptors for retroviruses that can recognize a variety of viruses with similar capsid (CA) geometry. Translating such properties in new antiviral compounds is challenging. New knowledge on IFN-induced and other restriction factors against FVs, and adaptive immune responses need to be tested in primary cells and in animals; they will be instrumental in understanding the outcomes of in vivo infection: persistence, latency and possibly clearance.

### 2.6. Foamy Viruses as Vectors for Gene Therapy

#### 2.6.1. Background and Review Talk

Foamy virus vectors (FVVs) are promising vectors for ex vivo gene therapy. They are characterized by an infectious DNA genome, a very large natural genome size, a very broad tropism and an apparent lack of pathogenic effect in natural hosts and humans infected through zoonosis. The session was reviewed by Dirk Lindemann. He provided a concise retrospective overview on the historical development of vectors based on different FV species, resulting in the current generation of FVV systems available. The general principles and characteristics of replication-competent and replication-deficient FVV systems were illustrated, and their major fields of preclinical applications were summarized. Furthermore, a synopsis of the features of two FVV systems developed by the Lindemann laboratory, one enabling pseudotyping of PFV vectors with heterologous glycoproteins [37] and another featuring transient genetic modification of target tissues by PFV mediated transfer of non-viral RNAs [38] was presented. The talk ended with an outlook to potential issues to be addressed next, which may ultimately enable a first use of FV-based vectors in a human clinical gene therapy trial.

#### 2.6.2. Summary of the Presentations

Fabian Lindel reported on the adaptation of the PFV-based non-viral RNA transfer system [38] mentioned above, for transient expression of CRISPR/Cas9 genome editing tools in target tissues. The results presented demonstrated efficient gene inactivation in cell lines achieved by a single application of this new PFV vector system. Furthermore, co-delivery of repair templates enabled specific editing of host cell genes, for example tagging of cellular gene by adding in-frame open reading frames (ORFs) encoding fluorescent proteins.

Nathan Sweeney briefly summarized his recently published results on pseudotyping PFV vector particles with SFVmac Env that reduced cytotoxic effects mediated by the PFV Env at high virus loads [39]. Such FVVs have recently been shown to transduce mesenchymal stromal cells more efficiently than alternative retroviral vectors. Furthermore, he presented work aimed at realizing the advantage of FVs having the largest mammalian retroviral genome and thus being expected to tolerate larger transgenes than other retroviral vectors. By inserting increasing lengths of DNA into an FVV, he determined the effect of transgene size on vector titer. Molecular assays showed that a 12 kb insert could be packaged, delivered and integrated into a target human cell genome at titers sufficient for ex vivo gene therapy. This greatly exceeds the maximum insert size of approximately 7 kb in lentivirus vectors, making FVVs an excellent option for the efficient and stable transfer of large transgenes.

#### 2.6.3. Perspectives

FVV have come of age. Well-characterized replication-deficient vectors based on different FV species are available and novel FVV for transient genetic modification or hybrid systems combining components of different retroviruses have been recently developed. FVV were shown to be effective in murine, canine and simian models of disease [40,41] and are awaiting their first application in a human gene therapy trial.

### 2.7. Foamy Virus Structural Studies

#### 2.7.1. Summary of the Presentations

The meeting provided new insights into FV structural studies concerning the RNase H and Gag proteins. The solution structure of the PFV RNase H domain has been solved previously [42]. It harbors a mixed five-stranded β-sheet, which is sandwiched by four α-helices and contains the so-called C-helix and a basic loop, which are important for positioning and binding of the nucleic acid substrate. In HIV-1 RNase H, this function is provided by a different loop located in the p51 subunit of the heterodimeric reverse transcriptase. Apart from this feature, the two RNase H domains exhibit a high degree of structural similarity. However, HIV-1 RNase H is very unstable in solution and thus it is not suitable for nuclear magnetic resonance (NMR) inhibitor studies. Birgitta Wöhrl showed how PFV RNase H can be used as a model to investigate the binding properties of new RNase H inhibitors directed against HIV RNase H [43]. NMR titration experiments with PFV RNase H and new RNase H inhibitors were used to determine the inhibitor binding site. Structural overlays and in silico docking analyses allowed the identification of the putative binding site in HIV-1 RNase H.

Despite little sequence homology, FV and orthoretroviral Gag proteins perform many equivalent functions, including genome packaging, virion assembly, trafficking and membrane targeting. However, in stark contrast to orthoretroviral Gag, there is a paucity of structural information for FV Gag, and it is unclear how these disparate molecules perform the same functions. Jonathan Stoye presented structural studies of the PFV Gag protein aimed at better understanding the FV Gag function and the structural relationship between FV- and orthoretroviral-Gag. Data was presented revealing how the dimeric N-terminal coiled-coil domain of PFV-Gag (PFV-Gag-NtD) is recruited by the Env leader peptide at the membrane through hydrophobic patches located on the periphery of the dimer “head domains” [44]. Notably, although PFV-Gag-NtD directed recruitment to the membrane is reminiscent of matrix (MA) function of orthoretroviral Gag, PFV-Gag-NtD shares no structural homology with MA, indicating that the structural basis of membrane targeting is an unrelated feature of FVs and orthoretroviruses. In addition, Jonathan Stoye presented further structural studies probing the functional overlap of FV and orthoretroviral Gag central regions. Although there is no sequence similarity in this region, the structures reveal significant structural homology between the central region of PFV Gag and archetypal orthoretroviral CA providing the first information that relates the Gag proteins of *Spuma-* and *Orthoretrovirinae*.

#### 2.7.2. Perspectives

So far, 3D structures of only a few FV proteins are available, namely the protease, RNase H, integrase and the N-terminal and central domains of Gag. Determining structures of additional FV proteins will shed light onto various mechanisms concerning the FV life cycle. These include, the structure of the complex of mature protease-reverse transcriptase (PR-RT) and PARM RNA to show how activation of the covalently linked PR is accomplished, the structure of full-length Gag protein to elucidate the mechanism of oligomerization as well as protein–protein and protein–RNA interactions, structures of the accessory proteins Tas and Bet which share no sequence homology with orthoretroviral accessory proteins and the structures of the envelope proteins, which would be invaluable to analyze the mechanism of FV uptake into cells.

### 2.8. Foamy Viruses: Novel Full-Length Virus Sequences and Reagents

#### Summary of the Presentations

This session was specially included in the meeting to address a critical gap in FV research regarding the generation of much needed reagents, assays, and sequence information. Arifa Khan presented data on the characterization of SFV type 3 strain FV2014, the first African green monkey isolate (ATCC VR-218) and currently designated as SFVcae-FV2014 according to the newly proposed taxonomy. The work included obtaining the full-length genome sequence assembly using high throughput sequencing, determining the phylogenetic relationship to other known SFVs and evaluating its biological properties based upon infectivity studies using different cell lines and host species. Piotr Kubiś presented molecular analysis of several bovine FV field isolates using nucleotide and amino-acid sequences in the signal peptide (SP) and surface glycoprotein (SU) regions of the *env* gene. DNAs isolated from virus-infected Cf2Th cells were PCR amplified and cloned for identification of sequence differences between American and Polish BFVs and sequence comparison was also done with different FVs. Caroline Lambert presented the construction of a BHK-21 indicator cell line for the quantification of gorilla SFVs, in which the U3 sequence of the LTR of zoonotic gorilla SFV (SFVggo-hu.BAK74) directs the expression of the β-galactosidase protein. Gorilla foamy virus activated β-galactosidase (GFAB) cells allowed efficient quantification of two zoonotic primary gorilla isolates and SFVs from three chimpanzee subspecies. Thus, this new SFV sensitive indicator cell line reveals the cross-transactivation of the SFVggo-hu.BAK74 LTR by gorilla and chimpanzee SFVs [45]. An additional feature of this session was discussion on a new proposed taxonomy and nomenclature for FVs. The final format is being prepared for publication.

## 3. Conclusions

As illustrated by the range of topics addressed above, studies of FVs lie within the forefront of current research in molecular virology, host–virus interactions, cell biology and human health: emerging infectious pathogens, microRNAs, retroelement integration, gene therapy and antiretroviral drugs. The quality of the presentations from several first attendees reflects the vitality of the scientific community working on FVs. We hope that the session summaries and highlights provided in this report will encourage interested researchers to join us at the 12th International Foamy Virus Conference, which is being planned for 2018 in Dresden, Germany.

## 4. Affiliations of Attendees

Aiewsakun, Pakorn pakorn.aiewsakun@zoo.ox.ac.ukUniversity of Oxford, Oxford, UKBuseyne, Florenceflorence.buseyne@pasteur.frInstitut Pasteur, Paris, FranceCao, Wenhu w.cao@dkfz.deGerman Cancer Research Center, Heidelberg, GermanyCouteaudier, Mathildemathilde.couteaudier@pasteur.frInstitut Pasteur, Paris, FranceDe Nys, Hélène helene.de-nys@ird.frIRD, Montpellier, FranceDjuicy, Deliadelia-doreen.djuicy@pasteur.frInstitut Pasteur, Paris/CIRFM Franceville, France/GabonGessain, Antoine antoine.gessain@pasteur.frInstitut Pasteur, Paris, FranceHu, Xiaomeihuxm189@163.comNankai University, Tianjin, ChinaKhan, Arifa arifa.khan@fda.hhs.govCBER/FDA, Silver Spring, MD, USAKubiś, Piotr kubip@piwet.pulawy.plNational Veterinary Research Institute, Pulawy, PolandKuźmak, Jacekjkuzmak@piwet.pulawy.plNational Veterinary Research Institute, Pulawy, PolandLambert, Caroline caroline.lambert@pasteur.frInstitut Pasteur, Paris, FranceLerner, Taga t.lerner@dkfz.deGerman Cancer Research Center, Heidelberg, GermanyLesage, Pascale pascale.lesage@inserm.frHôpital Saint-Louis, Paris, FranceLindel, Fabianfabian.lindel@tu-dresden.deTechnische Universität Dresden, GermanyLindemann, Dirk dirk.lindemann@tu-dresden.deTechnische Universität Dresden, GermanyLöchelt, Martin m.loechelt@dkfz.deGerman Cancer Research Center, Heidelberg, GermanyMaterniak-Kornas, Magdalena magdalena.materniak@piwet.pulawy.plNational Veterinary Research Institute, Pulawy, PolandMontange, Thomasthomas.montange@pasteur.frInstitut Pasteur, Paris, FranceQiao, Wentao wentaoqiao@nankai.edu.cnNankai University, Tianjin, China Richard, Léa lea.richard@pasteur.frInstitut Pasteur, Paris, FranceRichter, Stefanie stefanie.richter@googlemail.comTechnische Universität Dresden, GermanySantos, Andréandre.santos@ufrj.brUniversidade Federal do Rio de Janeiro, Rio de Janeiro, BrazilSimon, François francois.simon@sls.aphp.frHôpital Saint-Louis, Paris, FranceSoares, Marcelo masoares@biologia.ufrj.brUniversidade Federal do Rio de Janeiro, Rio de Janeiro, BrazilStoye, Jonathan jonathan.Stoye@crick.ac.ukFrancis Crick Institute, London, UKSweeney, Nathanns1410@imperial.ac.ukImperial College London, London, UKTaylor, Ian ian.taylor@crick.ac.ukFrancis Crick Institute, London, UKTeferedegne, Belete belete.teferedegne@fda.hhs.govCBER/FDA, Silver spring, MD, USATobaly-Papiero, Joëllejoelle.tapiero@univ-paris-diderot.frHôpital Saint-Louis, Paris, FranceWei, Guochao g.wei@dkfz.deGerman Cancer Research Center, Heidelberg, GermanyWöhrl, Birgitta birgitta.woehrl@uni-bayreuth.deUniversität Bayreuth, Bayreuth, GermanyXie, Yingpeng wentaoqiao@nankai.edu.cnNankai University, Tianjin, ChinaXu, Xiao wentaoqiao@nankai.edu.cnNankai University, Tianjin, ChinaZamborlini, Alessia alessia.zamborlini@univ-paris-diderot.frHôpital Saint-Louis, Paris, FranceZhang, Suzhen juantan@nankai.edu.cnNankai University, Tianjin, China

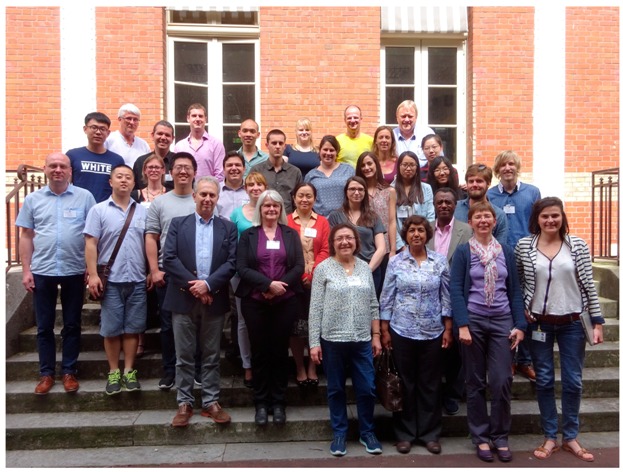



## Figures and Tables

**Table 1:** Trends in foamy virus (FV) research.

Exogenous FVs infect a wide range of mammals; the presence of endogenous FVs in the genome of several animal species suggest a possible ancient marine origin of theses retroviruses.
Zoonotic transmission of simian FVs has been reported all over the world and is currently ongoing; Bites are strongly associated with these transmission events; Neither pathogenicity nor human-to-human transmission have yet been reported.
The prototype foamy virus (PFV) integrase was the first retroviral integrase to be crystallized in complex with viral DNA; FV intasome interacts with nucleosomes.
The replication strategy of FVs shares features with orthoretroviruses, hepadnaviruses, and other retroid elements; Molecular biology of FVs has been mostly studied in cell culture systems. Replication in natural or experimental animal models is poorly described and probably diverse.
FVs induce type I interferons (IFNs), are susceptible to them and to type II IFNs, as well as to several restriction factors; with the exception of neutralizing antibodies against cell-free viral particles, adaptive immune responses are currently undescribed.
Foamy virus vectors (FVV) have come of age; Well-characterized replication-deficient vectors are available; FVV are effective in small and large preclinical models of human diseases.
3D structures of four FV proteins are known, namely the protease, RNAse H, integrase, and N-terminal Gag.

**Table 2:** Unanswered questions on foamy virus research.

What will be the next example of the early origins of FVs?
What are the first cells to be infected in naïve hosts and how does FV spread within an organism before becoming latent?
What are the anatomic sites of FV latent infection and active replication in vivo?
What are mechanisms of FV persistence?
What are the in vivo mechanisms of immune control of FV?
Is there any pathogenic effect of FVs, per se or in the context of co-infection with other pathogens?
What are the distribution and the magnitude of zoonotic infection in Africa, Asia, and America?
Are there biological abnormalities and/or clinical symptoms associated with FV infection in humans?
What are the roles of restriction factors on FV replication and transmissibility in the human infected hosts?
For how long will the FV receptor remain unknown?
When will the 3D structures of all FV proteins be available?
Do FV entry and early steps of replication impact cell tropism and host–virus interaction?
What are the respective roles of FV Gag and integrase in FV integration site selection?
What are the viral and cellular factors controlling FV gene expression and recovery from latency?
What are the role of FV-encoded microRNAs?
When will the first foamy virus vector be used in a human gene therapy trial?
